# The management of gynecological complications in long-term survivors after allogeneic hematopoietic cell transplantation—a single-center real-life experience

**DOI:** 10.1007/s00277-020-04034-1

**Published:** 2020-04-28

**Authors:** Łukasz Klasa, Alicja Sadowska-Klasa, Agnieszka Piekarska, Dariusz Wydra, Jan Maciej Zaucha

**Affiliations:** 1 Department of Gynecology, Oncological Gynecology and Gynecological Endocrinology, Medical University of Gdansk, Gdansk, Poland; 2 Department of Hematology and Transplantology, Medical University of Gdansk, Gdansk, Poland

**Keywords:** Gynecological GVHD, Hematopoietic cell transplantation, POI, Abnormal cytology

## Abstract

In everyday gynecological practice, there is an unmet need to manage survivors after allogeneic hematopoietic cell transplantation (allo-HCT). The major gynecological complications include premature ovarian insufficiency (POI), chronic graft-versus-host disease (cGVHD) of the anogenital zone (cGVHDgyn), and secondary neoplasms. Aiming to assess a real-life scale of problems associated with HCT, we performed a detailed analysis of a consecutive series of females after allo-HCT who were referred for a routine gynecological evaluation. The study includes 38 females after allo-HCT in whom gynecological examination with cervical smear and USG were performed, followed by colposcopy according to NCCN guidelines. NIH scoring system was used to classify a grade of cGVHDgyn. The incidence of cGVHD was 71% whereas GVHDgyn was 29%, including 5 patients with score 3 at the time of diagnosis. The other manifestations (frequently noted) included the skin, mucosa, eyes, and liver. Menopause was diagnosed in 93% females, and in 81% of them, POI criteria were fulfilled. Ovarian function resumed in 2 cases. The rate of abnormal cytology was 26%: 4 ASCUS, 1 AGUS, 1 LSIL, 3 HSIL/ASC-H, and one cytological suspicion of cervical cancer. GVHDgyn was documented in 10 patients, and 6 of them had abnormal cervical cytology. Early topical estrogen therapy led to a significant reduction in vaginal dryness (*p* < 0.05), dyspareunia (*p* < 0.05), and less frequent cGVHDgyn (*p* < 0.05). GVHDgyn develops in about 30% of long-term allo-HCT survivors. Topical estrogens and hormonal replacement therapy alleviate symptoms and prevent the occurrence of severe consequences of menopause.

## Introduction

The management of gynecological complications in long-term survivors after allogeneic hematopoietic cell transplantation (allo-HCT) remains still an unmet need in daily practice. Although the number of patients with severe symptoms is relatively small, they require special attention and comprehension. Allogeneic HCT affects the gynecological tract by different mechanisms. Gonadotoxicity of pre-transplant conditioning regimen leads to premature ovarian insufficiency (POI) in almost all adult women. Besides, the prolonged immunosuppression after transplantation increases the risk of secondary neoplasms [1, 2].

However, the most specific and often unrecognized complication is chronic graft-versus-host disease (cGVHD) of the anogenital zone (cGVHDgyn) that was reported for the first time in 1982 [3]. Donor-derived immunocompetent cells can become intolerant to host tissues and recognize them as their targets, leading to the development of a unique complication called graft-versus-host disease (GVHD) [4]. The acute form (aGVHD) that occurs typically up to 100 days post-allo-HCT does not affect the genitourinary tract. However, chronic GVHD which develops more than 50% of patients can involve every organ and tissue, including genital zone, with the symptomatology often mimicking autoimmune disorders. The diagnosis of cGVHD is based on the clinical manifestations and should be confirmed by histopathological examination in case of uncertainty. Chronic GVHD significantly affects the quality of life and remains the leading cause of late non-relapse mortality and morbidity after allo-HCT [5].

Aiming to establish a real-life scale of gynecological problems associated with allo-HCT, we performed a detailed analysis of a series of females after allo-HCT who were referred for a routine gynecological evaluation. We additionally discuss present practical recommendations for gynecological management, supported by the available literature.

## Materials and methods

Our study included 38 female patients who underwent allo-HCT at the University Clinical Centre in Gdansk between 2009 and 2018 and were referred in 2018 to the Department of Gynecology. Until 2018, the patients were under the care of local gynecologists. The basic characteristic of the study group is presented in Table 1. During routine visits, gynecological examination with cervical smear and USG were performed. In the case of abnormalities, colposcopy was done according to NCCN guidelines. From 2017, all patients received vaginal, topical estrogen therapy at the discharge from the Transplant Unit. Gynecological problems were categorized into three groups: cGVHD, menopause, and abnormal cervical cytology (Pap smear).

**Table 1:** The basic characteristic of the study group

Median age at HCT (range)	35.5 (16–58)
Diagnosis	
AML/MDS	25 (66%)
ALL	8 (21%)
Chronic myeloproliferative disorders	2 (5%)
Aplastic anemia	2 (5%)
Hodgkin’s disease	1 (2.5%)
Chemotherapy courses prior HCT:	Median (range)
Acute leukemias (intensive chemotherapy courses induction+consolidations)	4 (1–8)
MDS (no cht)/(5-aza)/induction	2pts/6 cycles (2–12)/1 (2pts)
Chronic myeloproliferative disorders	TKI/1 induction (blast crisis)
Aplastic anemia	No chemotherapy
Hodgkin’s disease	Prior autoHCT
Type of donor	
MSD	13 (34%)
MUD/MMUD	19 (50%)/5 (13%)
Haploidentical	1 (2.5%)
Type of conditioning regimen:	
TBI 12Gy-Cy	9 (24%)
Cy-Bu/Bu-Cy	20 (53%)
FluBu4/FluBu3	6 (16%)
Cy-ATG	2 (5%)
Other	1 (2.5%)
Median CD34x10^6^/kg	
PBSC	6.07 (range 4.27–8.36)
BM	1.2 (range 0.96–1.66)
aGVHD before cGVHD	18 (47%)
cGVHD	27 (71%)
Mild/moderate/severe	13/4/10
Dyspareunia:	
GVHDgyn	6/11 (54%)
No GVHDgyn	4/27 (15%)
Vaginal dryness:	
GVHDgyn	9/11 (82%)
No GVHDgyn	12/27 (44%)
Pregnancies/childbirth prior HCT	
0	11 (29%)/11 (29%)
1	7 (18%)/10 (26%)
2	14 (37%)/13 (34%)
More	6 (16%)/4 (11%)
Menopause prior HCT	6/38 (16%)
Menopause post HCT	30/32 (94%)

*AML* acute myeloid leukemia, *MDS* myelodysplastic syndrome, *ALL* acute lymphoblastic leukemia, *MSD* matched sibling donor, *MUD*/*MMUD* matched/mismatched unrelated donor, *PBSC* peripheral blood stem cells, *BM* bone marrow, *pts* patients, *TKI* tyrosine kinase inhibitors, *HCT* hematopoietic cell transplantation, *5-aza* azacitidie, *FluBu* fluradabine + busulfan, *CyBu* cyclophosphamide + busulfan, *TBI* total body irradiation, *ATG* anti-thymocyte globulin

Every patient was evaluated according to the National Institute of Health (NIH) Consensus criteria and scoring system of the genital cGVHD severity [6]. Reported symptoms and clinical signs in gynecological examination classify patient to 0–3 score (Table 2). Subjective symptoms are unspecific and may include dryness, burning, pruritus, dysuria, pain to touch, and dyspareunia leading to sexual dysfunction [7–14]. Particular features of genital cGVHD are lichen planus-like features, lichen sclerosus-like features, vaginal scarring, and clitoral/labial agglutination [6]. Other signs include patchy or generalized erythema, mucosal erosions and fissures, leukokeratosis, labial resorption, labial fusion, fibrinous vaginal adhesion, circumferential fibrous vaginal banding, vaginal shortening, and complete vaginal stenosis [6].

**Table 2:** Scoring of female genital cGVHD by NIH recommendations

	Score 0	Score 1	Score 2	Score 3
	No signs	Mild signs may have symptoms and discomfort at exam	Moderate signs may have symptoms and discomfort at exam	Severe signs, with or without symptoms
Signs	No	Erythema on mucosal surfaces, lichen planus-like, lichen sclerosus-like	Erosions in the vulvar mucosa, fissures in the vulvar folds	Labial fusion, clitoral/labial agglutination, fibrinous vagina adhesions, circumferential fibrous vaginal banding, vaginal shortening, and complete vaginal stenosis

The POI was defined as amenorrhea for at least 4 months with elevated levels of follicle-stimulating hormone > 40 IU/l in two measurements in 4–6 weeks intervals, in women before 40 years old [15, 16].

## Statistical analysis

Categorical variables were expressed as absolute numbers, and respective percentages and the differences between groups were compared using Pearson’s χ^2^ test. Continuous variables were expressed as median values with ranges. A *p* value of < 0.05 was considered statistically significant. All analyses were performed using STATISTICA version 13 (StatSoft, Inc.).

## Ethical approval

This study was performed in accordance with the latest version of the Declaration of Helsinki and received the approval of the Independent Bioethics Committee of the Medical University of Gdansk. All patients signed informed consent.

## Results

### Chronic GVHD

**Fig. 1:**
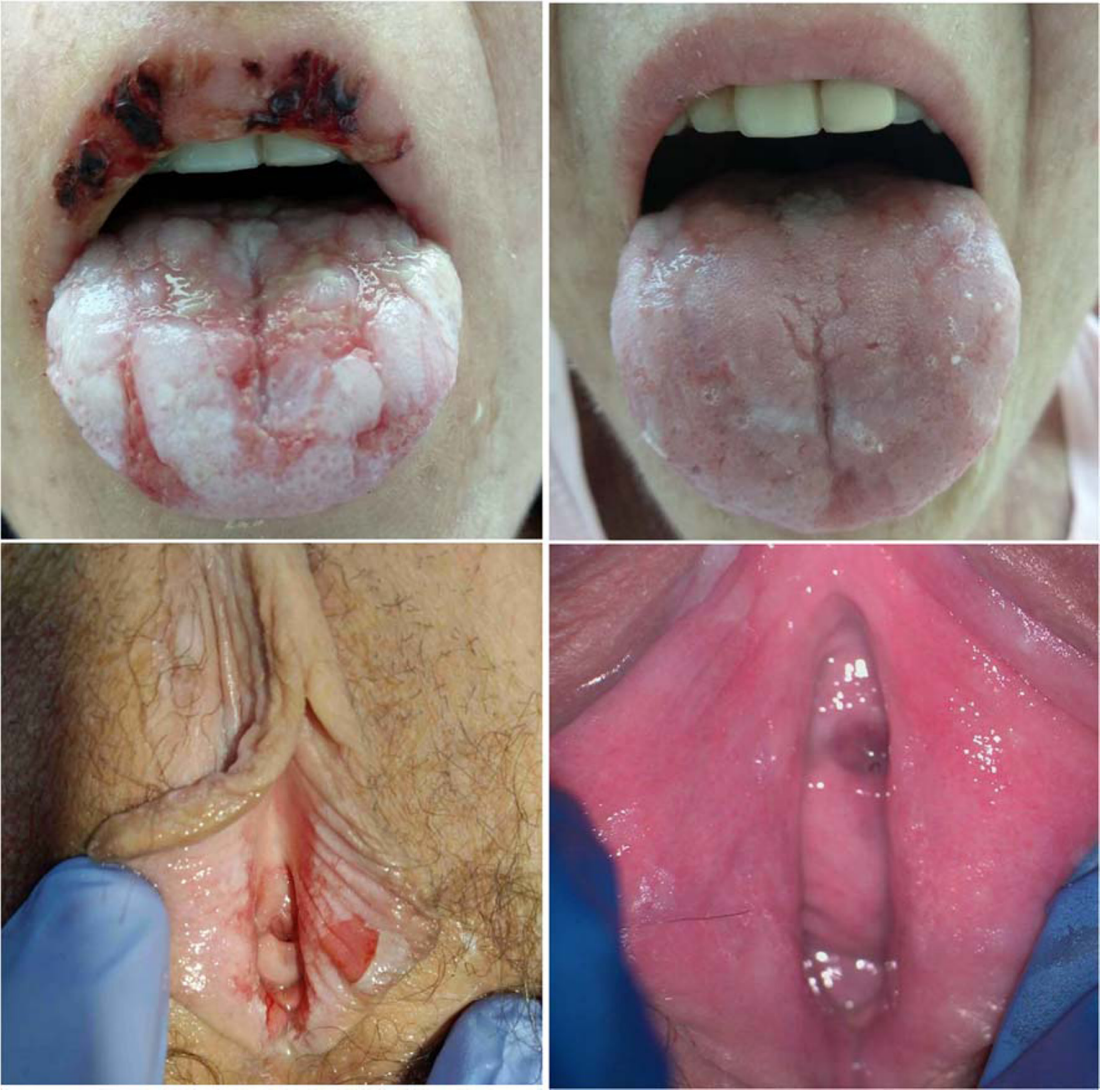
Simultaneous changes during cGVHD in the same patient: mucosal involvement and cGVHDgyn at the time of diagnosis (left side) and after 2 weeks of the treatment (right side)

In the study group, 71% (27/38) of referred patients suffered from cGVHD at some location (Fig. 1) with the most common presentation in the skin (18/27), liver (18/27), mucosa (17/27), and eyes (12/27). There was a statistically significant positive correlation between GVHDgyn and skin involvement (*p* < 0.01). The incidence of GVHDgyn was 29% (11 out of 38 patients) in all with cGVHD in other locations. Four patients had score 1, two score 2, and the rest (45%) score 3, which defines the severe form of cGVHD according to the NIH classification. There was one patient with partial and one with the complete vaginal stenosis. In two cases, circumferential fibrous bandings in vagina were found. We had a partial success in treatment of the patient with incomplete vaginal stenosis. Using concomitant surgery and steroid treatment, we managed to enlarge and lengthen vagina up to 8 cm enabling the patient to resume intercourse. We observed improvement in patients with milder forms of GVHD after treatment (Fig. 1), however not in a patient with complete vaginal stenosis.

### Menopause

Six women experienced already menopause before hematological treatment. From the remaining 32 patients (median age 33, range 16–47), menopause was diagnosed in 30 (94%) females. In 81% of them, the age at the time of menopause was below 40. Ovarian function resumed in 2 cases: in the patient with acute lymphoblastic lymphoma transplanted at the age of 17, and in one who underwent allo-HCT at the age of 16 for aplastic anemia. Fifty-five percent of women suffered from vaginal dryness, and 23% reported dyspareunia during the first gynecological visit. Twenty-one patients (55%) were sexually inactive after allo-HCT, due to gynecological symptoms (7 patients), a fear caused by inadequate information or permission (6 ones), a lack of a sexual partner (4 ones), and no prior sexual experience (4 ones). Seventeen patients received local estrogens at the discharge from the transplantation department. A significant reduction in vaginal dryness (*p* < 0.05) and dyspareunia (*p* < 0.05) as well as less frequent cGVHDgyn (*p* < 0.05) were observed in women with early topical estrogen therapy.

### Abnormal cytology

We found abnormal cytology in 10 women (26%): 4 ASCUS (atypical squamous cells of undetermined significance), 1 AGUS (atypical glandular cells of undetermined significance), 1 LSIL (low-grade squamous intraepithelial lesion), 3 HSIL/ASC-H (high-grade squamous intraepithelial lesion/ atypical squamous cells cannot exclude HSIL), and one cytological suspicion of cervical cancer. None of the patients had the HPV status checked. There were indications for the biopsy in 5 cases (Table 3). In the remaining 5 patients with abnormal cytology (LSIL, ASCUS) colposcopy revealed no pathological findings requiring a biopsy. In the histopathological examination, in case of uncertainty, immunohistochemical p16 staining was evaluated to confirm HPV infection. In 6 out of 10 patients with abnormal cervical cytology, the clinical signs of GVHDgyn were documented. With a median follow-up of 2.5 years (0.5–11 years), there was no case of cervical cancer documented in our group.

**Table 3:** Cytological and histopathological results in our observation

Cytology (Bethesda system)	Histopathology (biopsy)	Further management	Conclusion
Cytological suspicion of cervical cancer	cGVHD, no dysplasia	cGVHD topical treatment (steroids)	False positive cytology
ASC-H	CIN3(cervical intraepithelial neoplasia)	Cervical conization (CIN3 in removed tissue)	True positive cytology
ASC-H	No dysplasia (lichen sclerosus in the biopsy from the vulva)	Treatment of lichen sclerosus of the vulva (steroids)	False positive cytology
HSIL	No dysplasia	observation	False positive cytology
AGUS	Suspicion of CIN3 in cervical canal (insufficient material)	Cervical conization (no dysplasia in removed tissue)	False positive cytology

## Discussion

Our study shows that gynecological care provided by the local practitioners to the long-term allo-HCT female survivors was rather inadequate for the following findings. Firstly, despite the high incidence (71%) of cGVHD in 38 patients referred to our department, only 40% of them received any local treatment for gynecological symptoms. Secondly, 45% of patients had the highest (score 3) for female genital cGVHD according to the NIH recommendations, and even two patients were documented with partial or complete vaginal stenosis.

The higher cGVHDgyn morbidity in our group than reported by others [7, 14, 17, 18] (ranging between 25 and 60% depending on the criteria used) may result from some patient preselection since patients with gynecological symptoms were more likely to visit a dedicated gynecologist. High morbidity may also result from the peripheral blood stem cells used as a graft source in our patients, which is known to increase the risk of cGVHDgyn comparing with bone marrow transplantation grafts [7, 14]. Initially, morbidity rate for cGVHDgyn was estimated at only 3% [14]. The reason for such significant discrepancies was the lack of standard gynecological care after allo-HCT in many places, inducing our center, and the fact that patients rarely report symptoms at the time of routine post-transplant visits.

The median time of developing GVHDgyn after allo-HCT reported in the literature is 7–10 months [7, 14, 18]. In about 70% of patients, signs affect only the vulva, and in 30% both vulva and vagina, and vaginal symptoms usually appear after vulvar [14, 18]. We could not verify the time of GVHDgyn development in our group, due to suboptimal gynecological care in the past; however, from 2018 prospective analysis has been launched in our center. Chronic GVHDgyn is almost always associated with cGVHD manifestation in other organs such as the skin, mouth, liver, or gastrointestinal tract [7, 14, 18]. Zantomio et al. reported even 90% probability of GVHDgyn coexistence with other organ involvement, most often the mouth and skin [7]. The positive correlation between gynecological and skin manifestation of GVHD documented in our group corroborates this notion. Interestingly we found no statistically significant  correlation between GVHDgyn and mucosal manifestation. Although an isolated gynecological manifestation of GVHD is possible, we did not observe isolated cases in our study group [15]. The most common other manifestations of cGVHD in our series were also the skin, mouth, and liver. The National Institute of Health recommends gynecological examination in every woman after allo-HCT, especially when signs of cGVHD occur in the mouth mucosa, showing the strongest correlation with GVHDgyn [6]. Topical estrogen therapy should be initiated early after allo-HCT in all patients (even in those using hormonal replacement therapy; HRT) [7]. That may not prevent from GVHDgyn but minimalize menopausal symptoms mimicking GVHD and therefore helps to diagnose GVHD in an early stage. Due to a high probability of hypoestrogenism, all women after allo-HCT were advised to apply topical estrogen in the form of creams or vaginal globules twice a week from the time of discharge from the Transplant Unit. In our series, only 45% of patients complied with this recommendation. They presented, however, with fewer symptoms which indicate the protective role of local estrogen therapy in the prevention of dryness, dyspareunia, as well as may have some impact on GVHDgyn.

Topical glucocorticosteroids serve as an essential component in the treatment of GVHDgyn [7, 10, 14, 18]. Only some patients in our group received such treatment. Now we advise high vaginal application of hydrocortisone acetate 10 mg/g cream 1 g daily, for 4–6 weeks in GVHDgyn and local calcineurin inhibitors in case of more superficial lesions [7].

The risk of ovarian failure after allo-HCT depends mainly on the age at transplantation and conditioning regimen [1–4] (Table 4). High doses of alkylating agents and TBI irreversibly damage hormonal and reproductive function of the ovary in the majority of females [2, 4]. In less than 5% of women after allo-HCT, the ovarian function returns, but often for a short period, what was also observed in our study. In our group, resumption of ovarian function occurred solely in the youngest (< 20) allo-HCT recipients. According to Chiodi et al., age at allo-HCT and a dose of TBI have the strongest impact on the menopause. The cumulative probability of menstrual cycle resumption in patients aged < 20, 20–30, and > 30 years old was 88%, 27%, and only 3%, respectively. In turn, in females who received TBI and cyclophosphamide, the probability of regaining the ovarian function in the same ranges was 84%, 20%, and 0%, respectively [22]. Patients with a recovery of menstruation cycles are anyway at the high risk of infertility and early menopause [22]. Options of preservation and restoration of fertility can be categorized as well-established, debatable, and experimental. Embryo and egg freezing are well-established and effective options. Debatable ones include ovarian protection techniques such as gonadotropin-releasing hormone (GnRH) analogs, transposition of ovaries, or gonadal shields before radiation [19]. Reimplantation of cryopreserved ovarian tissue remains still experimental due to the unacceptable risk of potential recurrence of hematological malignancies [23]. Women with POI should be advised hormonal therapy of menopause (HTM) until the age of natural menopause if contraindications are excluded. There is no evident data on the optimal regimen, doses, and a route of sex hormones in POI [16]. The classical HTM or combined contraceptive pills may be used as replacement therapies, although HTM seems to have a better influence on the cardiovascular system and bone health [16]. Hormonal replacement therapy should be started early after transplantation in every woman after allo-HCT with POI under age of 40, even if asymptomatic [7, 14, 17, 24]. After 40 years old, HTM is recommended for patients with menopausal symptoms. The therapy should be continued until the age of natural menopause.

**Table 4:** A risk of therapy-induced gonadotoxicity and POI in young woman with hematological malignancies [19–21]

Risk of gonadotoxicity	Acute lymphoblastic leukemia (ALL)	Acute myeloid leukemia (AML)	Non-Hodgkin lymphoma (NHL)	Hodgkin lymphoma
Medium risk	Standard protocols		CHOP	ABVD
High risk	High dose of cyclophosphamide–multiple drug regimens		Radiotherapy: pelvis or abdomen Second line chemo: ICE, auto-HCT without TBI	Radiotherapy: pelvis or abdomen Chemotherapy: MOPP, BEACOPP Auto-HCT without TBI
Very high risk	Myeloablative conditioning regimens with TBI and/or high dose of alkylating agents preceding autologous or allogeneic HCT			
Unknown impact	Novel agents Blinatumomab Inotuzumab CAR-T cells	Novel agents Gemtuzumab ozogamicin FLT3 inhibitors	Novel agents ALK inhibitors BiTEs CAR-T cells	Novel agents Brentuximab vedotin Checkpoint inhibitors

A history of thromboembolic episodes is one of the contraindications of HTM. In some patients, a deep vein thrombosis occurs as a complication of the central catheter. It is a transient risk factor and in the absence of other contraindications should not be the reason for depriving of HTM. The transdermal route of estrogens in POI is probably the best option [14–16]. As estrogens avoid the first pass through the liver, the lower impact on coagulation cascade is observed [16]. Large observational studies have proved a lower risk of venous thromboembolism, and a stroke when a transdermal route of estrogen administration is used instead of an oral route. Moreover, the risk of thrombotic complications was not increased when compared with the age-matched controls in natural menopause [16].

A hepatic form of cGVHD and toxic liver damage poses another problem. In benign forms of liver impairment, the transdermal route of hormone administration is a safe option due to avoidance of liver circulation. In overweight or obese patients, percutaneous HTM administration is associated with a lower risk of thromboembolism in comparison with an oral route [15]. In our study group, no thrombotic events or hepatic toxicity of HTM was observed.

In our group, we did not observe secondary neoplasms which are well known, significant long-term complications of allo-HCT, and are responsible for 10% of late deaths [1, 25]. A large multicenter European study estimated the risk of secondary neoplasms to 11.5% after 15 years from allo-HCT, and the most common sites include the skin, oral cavity, larynx, cervix, and endometrium [26]. A prolonged immunosuppressive therapy in cGVHD leads to impaired T and B lymphocyte responses, leading to reactivation of latent HPV infections [2, 26, 27]. For this reason, cGVHD at any location promotes the process of cervical intraepithelial neoplasia [27, 28]. Moreover, a gynecological form of GVHD is an independent risk factor of HSIL [2]. The incidence of abnormal cytology in patients after allo-HCT reaches up to 40–70% according to different authors, which is many times higher than in a healthy population (4%) [2, 10, 14, 17, 18, 27]. We present similar data in our study. However, verification of abnormal cytology showed a high percentage of false positive cytology related to GVHDgyn, not yet reported in the literature. This observation indicates difficulties in cervical cancer prevention in allo-HCT recipients and a need for experienced gynecological assessment. This issue requires further investigation.

Complete vaginal stenosis in GVHDgyn diagnosed in one patient not only impairs the quality of life but also makes Pap smear difficult or even impossible to perform. Generally, cytology is recommended to be performed once a year in women after allo-HCT [2, 7, 10, 12, 14, 17, 27]. Histopathological verification should take place in centers experienced in recognizing cGVHD. Vaccination against HPV is advised in previously unvaccinated women < 26 years old [17, 27–29]. The immunization program should start 6–12 months after transplantation [17, 28, 29]. According to the guidelines of Infectious Diseases Working Group of Polish Adult Leukemia Group (PALG), three doses are recommended with 2-month intervals, although data on the immunogenicity of these vaccines in patients after allo-HCT is missing [30]. Older women who have not been vaccinated or patients vaccinated before can also benefit from additional vaccination after transplantation [29].

## Conclusions

The regular and multidisciplinary follow-up, including gynecological care, is essential to prevent serious complications after allo-HCT. Premature ovarian failure, irreversible in most cases, is one of the consequences of this treatment. The attending physician’s task is to make patients aware of the gonadotoxic effects of therapy and discuss fertility-preserving methods. Topical estrogens and HRT give relief of unpleasant symptoms and reduce the risk of severe complications of menopause in the bones, heart, or central nervous system. Chronic GVHD may also involve the urogenital zone. Since the symptoms of GVHDgyn are similar to those resulting from hypoestrogenism, patients should be encouraged to report any signs and to visit a gynecologist regularly. Only early diagnosis and prompt treatment of mild and moderate forms of GVHDgyn allow avoiding the most severe cases of complete vaginal stenosis with very limited therapeutic options left. Previous chemo- and radiotherapy, but mainly prolonged immunosuppressive therapy and GVHDgyn, increase the risk of secondary neoplasms significantly. Therefore, long-term allo-HCT survivors should remain under meticulous antineoplastic surveillance.
